# Supplementary material for: A Cross-Cultural Evaluation of Liking and Perception of Salted Butter Produced from Different Feed Systems
Source: Foods. 2020 Nov 28;9(12):1767. doi: 10.3390/foods9121767 (PMC7761244; doi:10.3390/foods9121767)
Supplement: Supplementary file 1 [file foods-09-01767-s001.pdf]

Table S1. Sensory terms for consumer study; in relation to Hedonics, Intensity Scales and for Just About Right assessment

**Hedonic**	**Scale**
Overall appearance	0= dislike extremely, 9= like extremely
Colour	0= dislike extremely, 9= like extremely
Flavour	0= dislike extremely, 9= like extremely
Salt	0= dislike extremely, 9= like extremely
Overall liking	0= dislike extremely, 9= like extremely
**Intensity Scale**	
Colour	0= low colour intensity, 9= high colour intensity
Flavour	0= low flavour intensity, 9= high flavour intensity
Salt	0= low salt intensity, 9= high salt intensity
Freshness	0= low freshness, 9= high freshness
Texture (firmness)	0= low firmness/ soft, 9= high firmness
**Just-About-Right**	
Colour	1= Much too light, 2= Too Light, 3= Just About Right, 4= Too Dark, 5= Much Too Dark
Flavour	1= Much too Low, 2=Too Low, 3= Just About Right, 4= Too Much, 5= Much Too Much

Saltiness	1= Much too little Salt, 2=Too Little Salt, 3= Just About Right, 4= Too Salty, 5= Much Too Salty
Texture	1= Much too soft, 2= Too soft, 3= Just About Right, 4= Too firm, 5= Much Too firm

Table S2.

Attribute list presented to panellists for Ranked Descriptive Analysis by assessors.

Descriptors	Intensity Scale	Definition
COLOUR		
Yellow Colour	0= very light, 9= very dark	Degree of lightness/ darkness of yellow colour
ODOUR		
Buttery	0= not detected, 9= extremely intense	Odour to that of butter
Milky	0= not detected, 9= extremely intense	Odour to that of milk
Grassy	0= not detected, 9= extremely intense	Odour to that of herbs, grass, forage
Rancid	0= not detected, 9= extremely intense	Odour to that of expired dairy products
FLAVOUR		
Salty	0= not detected, 9= extremely intense	Taste associated with NaCl
Sweey	0= not detected, 9= extremely intense	Taste associated with sucrose
Creamy	0= not detected, 9= extremely intense	Taste associated with cream / perception of fat in the mouth
Stale	0= not detected, 9= extremely intense	
Off Flavour	0= not detected, 9= extremely intense	
**TEXTURE**		
'Melt in mouth'	0= very slow, 9= very fast	Rate at which butter melts in the mouth
